# Photobiomodulation Dose–Response on Adipose-Derived Stem Cell Osteogenesis in 3D Cultures

**DOI:** 10.3390/ijms25179176

**Published:** 2024-08-23

**Authors:** Daniella Da Silva, Anine Crous, Heidi Abrahamse

**Affiliations:** Laser Research Centre, Faculty of Health Sciences, University of Johannesburg, Doornfontein, P.O. Box 17011, Johannesburg 2028, South Africa; 216040611@student.uj.ac.za (D.D.S.); acrous@uj.ac.za (A.C.)

**Keywords:** adipose-derived mesenchymal stem cells, differentiation inducers, three-dimensional cell culture, photobiomodulation, osteoblasts, osteoporosis regenerative therapy

## Abstract

Osteoporosis and other degenerative bone diseases pose significant challenges to global healthcare systems due to their prevalence and impact on quality of life. Current treatments often alleviate symptoms without fully restoring damaged bone tissue, highlighting the need for innovative approaches like stem cell therapy. Adipose-derived mesenchymal stem cells (ADMSCs) are particularly promising due to their accessibility, abundant supply, and strong differentiation potential. However, ADMSCs tend to favor adipogenic pathways, necessitating the use of differentiation inducers (DIs), three-dimensional (3D) hydrogel environments, and photobiomodulation (PBM) to achieve targeted osteogenic differentiation. This study investigated the combined effects of osteogenic DIs, a fast-dextran hydrogel matrix, and PBM at specific wavelengths and fluences on the proliferation and differentiation of immortalized ADMSCs into osteoblasts. Near-infrared (NIR) and green (G) light, as well as their combination, were used with fluences of 3 J/cm^2^, 5 J/cm^2^, and 7 J/cm^2^. The results showed statistically significant increases in alkaline phosphatase levels, a marker of osteogenic differentiation, with G light at 7 J/cm^2^ demonstrating the most substantial impact on ADMSC differentiation. Calcium deposits, visualized by Alizarin red S staining, appeared as early as 24 h post-treatment in PBM groups, suggesting accelerated osteogenic differentiation. ATP luminescence assays indicated increased proliferation in all experimental groups, particularly with NIR and NIR-G light at 3 J/cm^2^ and 5 J/cm^2^. MTT viability and LDH membrane permeability assays confirmed enhanced cell viability and stable cell health, respectively. In conclusion, PBM significantly influences the differentiation and proliferation of hydrogel-embedded immortalized ADMSCs into osteoblast-like cells, with G light at 7 J/cm^2^ being particularly effective. These findings support the combined use of 3D hydrogel matrices and PBM as a promising approach in regenerative medicine, potentially leading to innovative treatments for degenerative bone diseases.

## 1. Introduction

Osteoporosis and other degenerative bone diseases present significant challenges to healthcare systems worldwide, largely due to their prevalence and their severe impact on quality of life [1]. These conditions not only increase the risk of fractures and reduce mobility, but also place a substantial burden on healthcare resources, especially within aging populations [2]. The impact of these diseases is further highlighted by their contribution to increased morbidity, mortality, and economic strain on both individuals and healthcare systems [3]. Current treatment approaches primarily focus on alleviating symptoms rather than fully restoring damaged bone tissue, underscoring the urgent need for more effective therapeutic strategies [4]. This need for innovative solutions has driven interest in regenerative medicine, particularly the use of stem cells (SCs) to transform treatment approaches for various diseases and injuries [5]. Stem cells are central to these advancements due to their ability to differentiate into specific cell types and support tissue repair and regeneration [6]. Among the various types of stem cells, adipose-derived mesenchymal stem cells (ADMSCs) stand out as particularly promising because of their accessibility, abundant supply, and strong differentiation potential [7]. Compared to other stem cells, such as those derived from bone marrow, ADMSCs offer practical advantages, including less invasive harvesting procedures and the ability to obtain larger quantities, making them more suitable for clinical applications [8]. However, ADMSCs have a natural tendency to differentiate into adipose tissue, which necessitates the use of differentiation inducers (DIs) to guide them toward osteogenic pathways [9]. This ease of access and ability to be directed toward specific lineages justify their use in studies focused on bone regeneration. Despite the use of DIs, ADMSCs may still favor adipogenic differentiation, highlighting the need to supplement DIs with biomechanical and biophysical stimuli, such as a three-dimensional (3D) hydrogel environment and photobiomodulation (PBM), to achieve precise lineage-specific differentiation [10]. The rationale for this combination is to create a microenvironment that closely mimics the natural bone niche [11]. While DIs can initiate osteogenic differentiation, their effectiveness is often limited by the propensity of ADMSCs to revert to their adipogenic lineage, particularly in the absence of additional cues [12]. This limitation underscores the importance of integrating other factors, such as the structural support provided by 3D hydrogels, which promote cell–cell interactions and tissue-like organization [13], and the biostimulatory effects of PBM, which enhance cellular activity and differentiation [14]. By addressing these limitations, the combination of DIs, 3D hydrogels, and PBM ensures more effective and targeted osteogenic differentiation, thereby improving the potential for successful bone regeneration. Three-dimensional cell culture, in particular, enhances ADMSC differentiation into osteoblasts by providing a physiological-like microenvironment [15]. This approach effectively bridges the gap between in vitro cell culture and in vivo animal models, offering more accurate data on cell interactions and advancing stem cell research [16]. Photobiomodulation (PBM) is well documented for its ability to enhance cell proliferation and differentiation [17]. When applied to ADMSCs, PBM has been shown to boost their growth and differentiation into various cell types [18]. The effectiveness of PBM on ADMSCs is highly dependent on key factors such as wavelength and fluence, which can either stimulate or inhibit cellular responses [19]. In this study, we selected wavelengths between 660–850 nm and fluences between 3–10 J/cm^2^ to enhance cellular proliferation, while wavelengths of 495–570 nm were chosen to promote cellular differentiation [20]. These specific wavelengths were selected based on their proven ability to elicit the desired cellular responses, with longer wavelengths providing deeper tissue penetration and shorter wavelengths being more effective for surface-level stimulation [21]. The role of fluence is particularly crucial due to the biphasic dose–response phenomenon, where low-to-moderate doses of PBM stimulate cellular activity, while higher doses can have an inhibitory or even detrimental effect [22]. This emphasizes the importance of carefully calibrating PBM parameters to optimize therapeutic outcomes. Standardizing PBM methodologies has significant clinical potential. Consistent protocols could establish PBM as a reliable adjunctive therapy in regenerative medicine, particularly for stem cell treatments [23]. Such standardization could lead to more predictable and effective interventions for conditions like degenerative bone diseases, where enhancing stem cell proliferation and differentiation is critical for successful tissue regeneration. Moreover, establishing standardized PBM protocols could facilitate broader adoption in clinical practice, potentially improving patient outcomes and advancing the field of regenerative medicine [24,25,26,27,28].

This in vitro study investigated the combined effects of osteogenic DIs, a fast-dextran hydrogel matrix, and PBM at specific wavelengths and fluences on the proliferation and differentiation of immortalized ADMSCs into osteoblasts. To ensure the relevance and applicability of our findings to in vivo conditions and potential clinical treatments, we carefully selected experimental parameters that closely mimicked the physiological environment. The fast-dextran hydrogel matrix was chosen for its ability to replicate the extracellular matrix and in providing a scaffold that supports cell growth and differentiation in a manner similar to in vivo bone tissue. Additionally, the PBM wavelengths and fluences were selected based on their proven efficacy in promoting osteogenic differentiation in previous studies, aligning with conditions that could be realistically applied in clinical settings. By combining these elements, we aimed to bridge the gap between in vitro models and potential in vivo applications, thereby enhancing the translatability of our findings to real-world therapeutic strategies.

## 2. Results

### 2.1. Alkaline Phosphatase Detection

**Figure 1:**
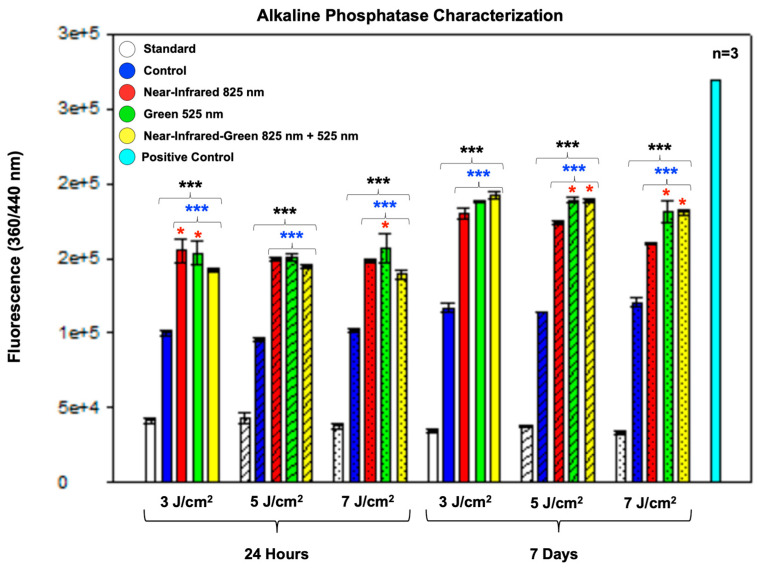
The detection of alkaline phosphatase levels in differentiated immortalized adipose-derived mesenchymal stem cells was measured at 24 h and 7 days post-photobiomodulation. At 24 h, a statistically significant increase in ALP levels was observed in the control group compared to the standard. Additionally, all experimental groups showed a statistically significant increases in ALP levels compared to both the standard and control groups. Specifically, the G wavelength at 3 J/cm^2^ and 7 J/cm^2^ exhibited the most significant increase in ALP levels among the experimental groups. At 7 days, there was a notable rise in ALP levels in the control group compared to the standard group, and all experimental groups showed significant increases compared to the standard and control groups. Moreover, the G and NIR-G experimental PBM groups demonstrated an overall increase in ALP levels across 5 J/cm^2^ and 7 J/cm^2^ fluences at 7 days post-treatment. The data are expressed as mean ± SE. * *p* < 0.05, *** *p* < 0.001. Black stars (*) indicate comparisons between the specified samples and the standard group. Blue stars (*) denote comparisons between the experimental samples and the control group. Comparisons among the experimental PBM groups are marked with red stars (*). The sample size was n = 3.

Alkaline phosphatase (ALP) is a membrane-bound glycoprotein known as an early osteogenic marker of bone formation and calcification, and it is used to determine the osteogenic differentiation of cells. The ALP detection assay identified statistically significant (*p* < 0.001) increases in ALP levels in the control group compared to the standard group, as well as in all experimental groups (*p* < 0.001) compared to both the standard and control groups at 24 h and 7 days post-photobiomodulation (PBM) treatment. Additionally, at 24 h post-treatment, the NIR and G wavelengths at 3 J/cm^2^, and the G wavelength at 7 J/cm^2^, showed statistically significant (*p* < 0.05) increases in ALP levels compared to each other. At 7 days post-treatment, statistically significant (*p* < 0.05) increases in ALP levels were observed in the G and NIR-G wavelengths at 5 J/cm^2^ and 7 J/cm^2^ compared to the other experimental groups (Figure 1).

### 2.2. Evaluated Calcium Deposition

**Figure 2:**
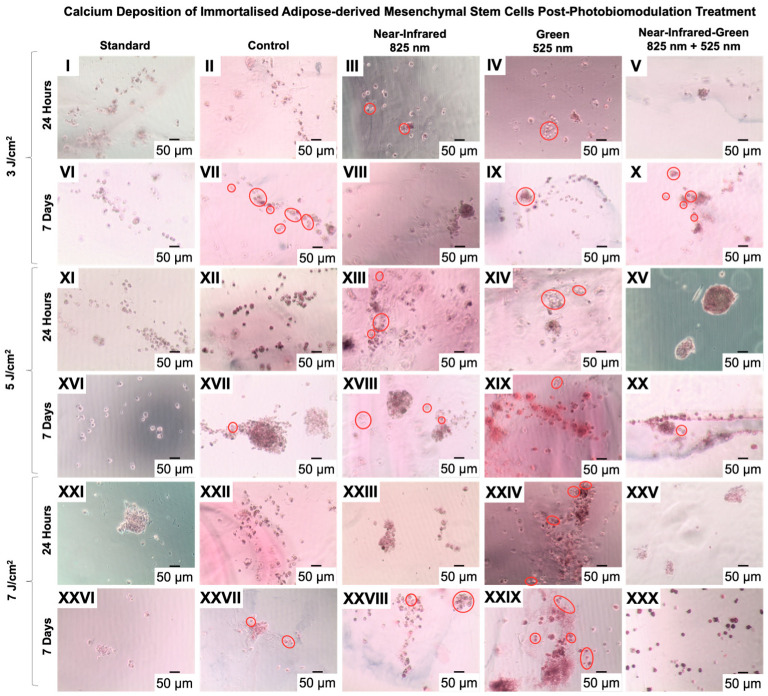
Morphological characterization of adipose-derived mesenchymal stem cells using Alizarin red S staining. The observation of vibrant orange to red deposits at both 24 h and 7 days post-photobiomodulation treatment indicates calcium deposition, suggesting potential osteogenic differentiation (III, IV, VII, IX, X, XIII, XIV, XVII, XVIII, XIX, XX, XXIV, XXVII, XXVIII, and XXIX). (10× magnification and 50 μm scale bar).

Alizarin red S staining has long served as a method to assess calcium-rich deposits by cultured cells. These deposits appear as vibrant orange hues in morphological analysis. In our study, cells cultured within a dextran hydrogel matrix and osteogenic induction media and stained with Alizarin red S displayed distinct bright orange to red calcium nodules. This observation was notable as early as 24 h after PBM treatment in experimental groups exposed to NIR and G wavelengths at fluences of 3 J/cm^2^ and 5 J/cm^2^, as well as in the G group at 7 J/cm^2^ (Figure 2 III, IV, XIII, XIV, and XXIV). The early onset of calcium deposition exclusively in the PBM-treated groups suggests PBM’s potential to accelerate ADMSC differentiation into osteogenic lineage cells, particularly at the G 525 nm wavelength and 7 J/cm^2^ fluency, which was evident from the visibly increased calcium deposits. By Day 7 post-PBM treatment, calcium deposition was evident at 3 J/cm^2^ fluency in the G and NIR-G groups (Figure 2 IX and X); at 5 J/cm^2^ in the NIR, G, and NIR-G groups (Figure 2 XVIII, XIX, and XX); and at 7 J/cm^2^ in the NIR and G groups (Figure 2 XXVIII and XXIX). Additionally, calcium nodules were observed in the control group by Day 7, indicating the synergistic role of the osteogenic differentiation inducer cocktail and hydrogel disc in guiding and influencing cell differentiation.

### 2.3. Quantification of Cellular Proliferation via Adenosine Triphosphate Detection

**Figure 3:**
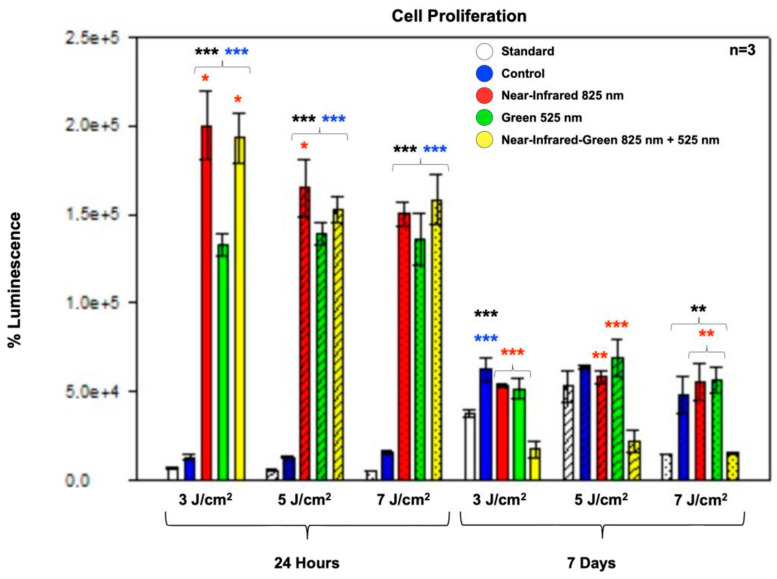
Cell proliferation: ATP luminescence assay of the differentiated immortalized adipose-derived mesenchymal stem cells’ ATP levels measured at 24 h and 7 days post-photobiomodulation. At 24 h, all experimental groups showed a statistically significant increase in ATP levels compared to the standard and control groups. At 7 days, a significant rise in ATP levels was observed in the control, NIR, and G PBM groups compared to the standard at fluences of 3 J/cm^2^ and 7 J/cm^2^. However, the NIR-G experimental PBM group exhibited an overall decline in ATP levels across all three fluences at 7 days post-treatment. The data are presented as mean ± SE. Significance levels are denoted as follows: * *p* < 0.05, ** *p* < 0.01, *** *p* < 0.001. Black stars (*) represent comparisons between the specified samples and the standard group, while blue stars (*) indicate comparisons between the experimental samples and the control group. Comparisons among the experimental PBM groups are marked with red stars (*). The sample size was n = 3.

The ATP luminescence assay was employed to assess the combined effects of the hydrogel matrix, osteogenic induction medium, and PBM irradiation on the proliferation of immortalized ADMSCs and their interdependent relationship with metabolism. This assay generates a luminescent signal through the luciferase enzyme, and it is directly proportional to the ATP concentration in the sample. Higher ATP concentrations indicate increased mitochondrial stimulation and cellular proliferation. The ATP luminescence assay results (Figure 3) revealed a significant (*p* < 0.001) elevation in ATP levels across all experimental PBM groups compared to the standard and control groups at 24 h. Notably, the NIR and NIR-G wavelengths at 3 J/cm^2^ and the NIR wavelength at 5 J/cm^2^ exhibited the most significant (*p* < 0.05) proliferation increases when compared to each other at 24 h post-treatment. At 7 days post-PBM treatment, proliferation significantly increased in the NIR and G groups at 3 J/cm^2^ (*p* < 0.001); in the NIR (*p* < 0.01) and G (*p* < 0.001) groups at 5 J/cm^2^; and in the NIR and G groups at 7 J/cm^2^ (*p* < 0.01) when compared to each other. However, overall proliferation decreased in all experimental PBM groups at 7 days compared to the levels observed at 24 h, particularly at 3 J/cm^2^ fluency. This decline suggests a redirection of ATP resources toward cell differentiation rather than proliferation [29].

### 2.4. Assessed Cellular Viability

**Figure 4:**
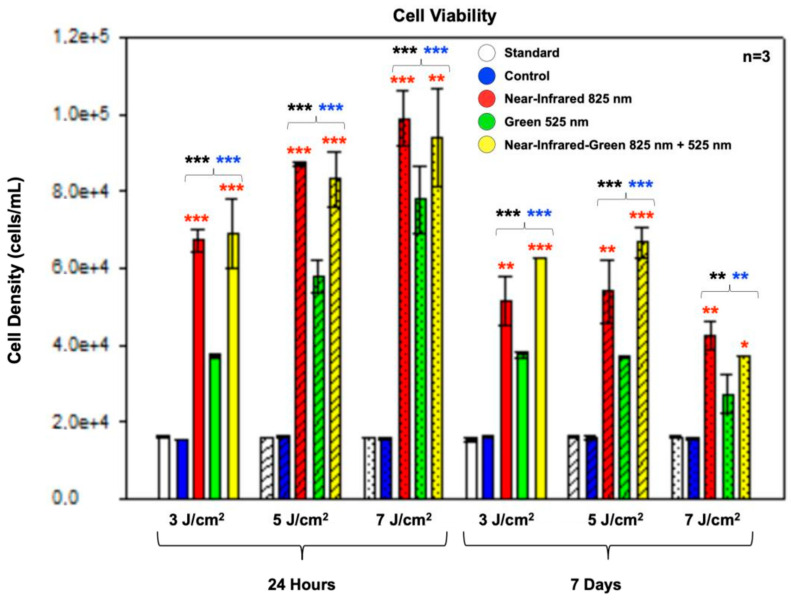
Cell viability analysis of immortalized adipose-derived mesenchymal stem cells at 24 h and 7 days post-photobiomodulation treatment. The MTT assay revealed a statistically significant increase in cell viability across all experimental groups at both time points compared to the standard and control groups. Additionally, the NIR and NIR-G experimental groups showed statistically significant increases in cell viability compared to the G experimental group at all three fluences, both at 24 h and 7 days post-treatment. The data are shown as mean ± SE. Significance levels are indicated by * *p* < 0.05, ** *p* < 0.01, and *** *p* < 0.001. Black stars (*) denote comparisons between the specified samples and the standard group, blue stars (*) show comparisons between the experimental samples and the control group, and red stars (*) indicate comparisons among the experimental PBM groups. The sample size is n = 3.

The MTT assay was used to evaluate cellular viability by measuring metabolic activity. This colorimetric method relied on metabolically active cells converting the yellow tetrazolium salt, MTT, into purple formazan crystals, which were then dissolved to produce a measurable colored solution. The MTT assay (Figure 4) revealed statistically significant increases in the cell viability (*p* < 0.001) among all experimental groups at both 24 h and 7 days post-PBM treatment compared to the standard and control groups. Additional significant differences were observed among the cells treated with NIR and NIR-G PBM wavelengths (*p* < 0.001) at 24 h, as well as between NIR (*p* < 0.01) and NIR-G at 3 J/cm^2^, 5 J/cm^2^ (*p* < 0.001), and 7 J/cm^2^ (*p* < 0.05) fluences at day 7 post-treatment. However, the noticeable decline in cell viability at 7 days compared to 24 h post-PBM treatment did not imply negative effects on cell health due to PBM. This decline may be attributed to the increased cell proliferation leading to competition for resources within the culture medium [30].

### 2.5. Lactate Dehydrogenase-Based Cell Membrane Permeability Analysis

**Figure 5:**
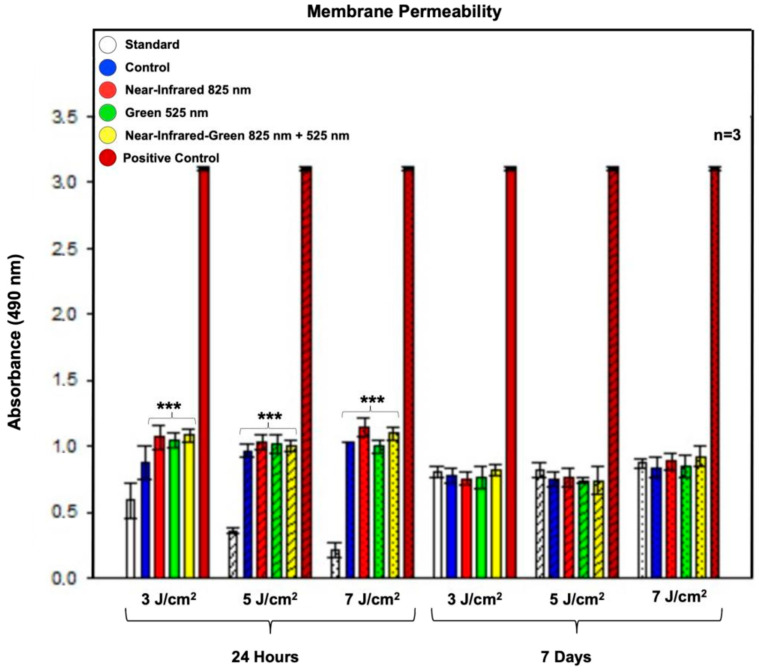
Membrane permeability analysis: The lactate dehydrogenase cytotoxicity assay indicated a significant increase in LDH leakage at 24 h post-PBM treatment in both the control and all experimental groups compared to the standard cell group. Importantly, despite the observed increase in LDH leakage, it did not lead to cell fatality, as evidenced by a comparison with the experimental cytotoxic positive control. The data are presented as mean ± SE. Significance levels are marked as follows: *** *p* < 0.001. Black stars (*) indicate comparisons between the specified samples and the standard group. The sample size is n = 3.

If a cell membrane is damaged, LDH leaks into the media, indicating cytotoxicity or membrane permeability damage. The LDH results (Figure 5) showed a significant increase (*p* < 0.001) in LDH leakage in both the control and all experimental groups at 24 h post-PBM treatment compared to the standard group. However, despite this notable increase, the levels did not reach toxic concentrations when compared to the cytotoxic positive control, which represented a hundred percent cell toxicity and cell death. No significant increase in LDH leakage was observed at 7 days post-PBM treatment. The relatively stable cell health indicated by the MTT viability assay over the 7-day experimental period suggests that the increased LDH levels were not due to the plasma membrane damage induced by PBM [31]. The slight LDH leakage observed may be attributed to changes in cell membrane permeability, possibly due to differentiation [32], or due to contact inhibition and nutrient depletion within the cell culture medium after an extended period without a medium change [30].

## 3. Discussion

To compare the findings of the present study with previous research on PBM and its effects on osteogenic differentiation, we focused on key experimental conditions, cell types, and PBM parameters. Our investigation centered on the combined effects of osteogenic DIs, a fast-dextran hydrogel matrix, and PBM at specific wavelengths and fluences on the proliferation and differentiation of immortalized ADMSCs into osteoblasts. The decision to use ADMSCs was driven by their accessibility, abundant supply, and strong differentiation potential, making them particularly suitable for our study compared to other stem cell types. 

In this study, ALP activity served as an early marker for osteogenic differentiation, and it was found to be consistent with the findings of Peng et al. and Oliveira et al., who also reported significant increases in ALP levels following PBM treatment [33,34]. Our results revealed robust ALP elevation across all experimental groups compared to the standard and control groups, indicating enhanced osteogenic potential. Specifically, the role of NIR light in bone regeneration, highlighted by Peng et al., aligns with our findings of NIR’s efficacy in stimulating ALP activity, particularly at lower fluences [33]. Additionally, the distinct enhancement of ALP levels with G light at higher fluences observed in our study echoes the wavelength-specific effects on osteogenesis reported by Yaralı Çevik et al. [35].

Calcium deposition, assessed through Alizarin red S staining, corroborated the findings from studies by Jiang et al. and Diniz et al., demonstrating early and substantial calcium nodule formation in PBM-treated groups [36,37]. Our results similarly showed accelerated calcium deposition as early as 24 h post-treatment, particularly under NIR and G wavelengths, indicating PBM’s role in promoting osteogenic differentiation in ADMSCs within a three-dimensional hydrogel matrix. This is consistent with observations of enhanced osteogenic outcomes in scaffold-free microtissues by Yaralı Çevik et al., suggesting that PBM facilitates early mineralization processes crucial for bone tissue engineering applications [35].

The ATP luminescence assay, which was used to assess cell proliferation and metabolic activity, revealed significant increases in ATP levels across all experimental groups post-PBM treatment, which is indicative of heightened cellular metabolic activity and proliferation potential. These findings parallel those of Zaccara et al. and Adolpho et al., where PBM enhanced cell viability and proliferation rates, which is crucial for tissue regeneration processes [38,39]. Our study noted a shift in ATP dynamics over time, with initial proliferation peaks at 24 h followed by a decline at 7 days, suggesting a transition from proliferation to differentiation phases influenced by PBM, as observed in other studies evaluating DPSCs and MSCs under similar conditions.

The MTT assay results assessing cell viability demonstrated sustained metabolic activity and cell health across experimental groups, contrasting with transient increases in LDH leakage observed at 24 h post-treatment, which normalized by Day 7. This finding aligns with observations by Tsai et al. regarding membrane integrity and cell viability under PBM, emphasizing the benign nature of PBM-induced changes in membrane permeability and metabolic responses, which are crucial for long-term cell viability and functionality [40].

Furthermore, our study’s comparison with studies using innovative scaffold materials, such as P(VDF-TrFE)/BaTiO3 by Adolpho et al. and injectable hydrogels loaded with rhBMP4 by Diniz et al., underscores PBM’s versatility across different scaffold systems and stem cell types in enhancing osteogenic differentiation [36,39]. Our findings extend this understanding by highlighting the synergistic effects of PBM with specific wavelengths and fluences on ADMSCs embedded within a dextran hydrogel matrix, elucidating the optimal conditions for promoting early osteogenic markers and mineralization events critical for bone tissue engineering.

However, there are limitations to this study that must be acknowledged. While our in vitro findings offer significant insights into the potential of PBM-enhanced osteogenic differentiation, the controlled conditions of the laboratory environment do not fully replicate the complexities of in vivo systems. The translation of these findings to clinical settings requires further investigation to validate the efficacy and safety of these approaches. Additionally, the variability in the PBM response due to differences in light sources and fluence parameters underscores the need for standardized protocols to ensure consistent therapeutic outcomes. Future research should focus on optimizing PBM parameters for ADMSC differentiation and viability, exploring the effects of different dextran hydrogel stiffnesses and advancing the understanding of molecular mechanisms underlying PBM’s effects on stem cells. Identifying the key signaling pathways and genetic markers involved in PBM-induced differentiation and proliferation will provide deeper insights into its therapeutic potential and help refine treatment protocols.

This study advances strategies for enhancing osteogenic differentiation and tissue regeneration that is consistent with prior research, and it highlights PBM’s potential as a non-invasive tool in regenerative medicine. However, the limitations mentioned underscore the need for ongoing research to bridge the gap between in vitro and in vivo models, ensuring the clinical applicability of PBM-enhanced stem cell therapies.

## 4. Materials and Methods

### 4.1. Cell Propagation 

Immortalized ADMSCs (ASC52telo hTERT, ATCC^®^ SCRC-4000™) were cultured in an induction medium consisting of Dulbecco’s Modified Eagle’s Media (DMEM) (Sigma-Aldrich^®^, St. Louis, MO, USA, D5796) supplemented with 10% fetal bovine serum (FBS Superior) (Biochrom, Holliston, MA, USA, S0615) and 1% Penicillin-Streptomycin-Amphotericin B solution (Sigma-Aldrich^®^, P4333/A2942). The cells were maintained in Nunc™ EasYFlask™ 75 cm^2^ cell culture flasks (ThermoScientific™, Waltham, MA, USA, 156499) and incubated at 37 °C with 5% CO_2_ and 85% humidity (Heracell™ 150i CO_2_ Incubator, ThermoScientific™, 51026280). They were seeded at a density of 1 × 10^4^ cells/mL in BRAND^®^ 96-well strip plates (Sigma-Aldrich^®^, BR782306) with osteogenic induction media containing [50 nM] dexamethasone, [10 mM] β-glycerol phosphate disodium, and [0.2 mM] ascorbic acid as differentiation inducers. Additionally, a 10 μL fast-dextran hydrogel disc (Sigma-Aldrich^®^, TRUE3-1KT) was added to the culture. Before irradiation, the cells were incubated for 7 days in the osteogenic induction media whilst embedded within the dextran hydrogel disc.

### 4.2. Application of Photobiomodulation to Cells

The experiment was structured into five groups: Standard, with cells encapsulated in dextran hydrogel but without DIs or PBM treatment; Control, with cells in hydrogel receiving DIs but no PBM treatment; NIR, where cells in hydrogel received DIs and PBM at 825 nm with fluences of 3 J/cm^2^, 5 J/cm^2^, or 7 J/cm^2^; G, where the cells in hydrogel received DIs and PBM at 525 nm with the fluences mentioned; and NIR-G, involving cells embedded within a hydrogel disc receiving DIs and PBM at combined wavelengths of 825 nm and 525 nm at 3 J/cm^2^, 5 J/cm^2^, or 7 J/cm^2^. Before irradiation, 100 µL of a cell culture medium was added to designated wells in each group. The cells were then irradiated using a NIR 825 nm Diode Laser (National Laser Centre of South Africa, SN 101080908ADR-1800), a G 525 nm Diode Laser (National Laser Centre of South Africa, EN 60825-1:2007) [41], and combined NIR-G wavelengths (825 nm and 525 nm) at the specified fluences. Laser power output (mW) was measured with a FieldMate Laser Power Meter (Coherent, Saxonburg, PA, USA, 1098297), and the irradiation time, based on fluence, was determined using a High-Sensitivity Thermopile Sensor PM3 (Coherent, 1098336) [42,43]. Detailed laser parameters are provided in Table 1.

The calculation for the duration of irradiation was determined using the formula given in Equation (1):

(1) $$\begin{array}{c} mW/{cm}^{2}=\frac{mW}{\pi \times {(r}^{2})},\\ W/{cm}^{2}=\frac{mW/{cm}^{2}}{1000},\\ Time\left(s\right)=\frac{J/{cm}^{2}}{W/{cm}^{2}}. \end{array}$$

The above equation details the duration of the laser irradiation, where mW/cm^2^ denotes power density, W/cm^2^ indicates intensity, and s denotes exposure time.

### 4.3. Detection of Alkaline Phosphatase

During the osteogenic differentiation process, alkaline phosphatase (ALP) activity is a key marker for the transition of MSCs into osteoblasts. For the ALP activity assay, cells were first cultured under osteogenic conditions. After reaching the appropriate differentiation stage, cells were lysed to obtain non-secreted ALP. Specifically, 10 μL of 10X lysis solution (Promega, Madison, WI, USA, G1780) was added per 100 μL of cell suspension, followed by a 45 min incubation at 37 °C. To prepare the substrate solution for ALP detection, 1 mg of 4-methylumbelliferyl phosphate disodium salt was dissolved in 330 μL of deionized water to achieve a 10 mM concentration. Prior to the assay, all buffers were thawed to room temperature. The assay was conducted using a 96-well microplate (Sigma-Aldrich^®^, BR782306), where 20 μL of each sample, including a negative control, was placed into each well. Samples were incubated at 65 °C for 30 min to reduce background and nonspecific phosphatase activity, followed by rapid cooling on ice for 2 min. Subsequently, 20 μL of Dilution Buffer and 160 μL of Fluorescent Assay Buffer were added to each well. Finally, 1 μL of the 10 mM substrate solution was introduced, and the mixture was thoroughly homogenized using a wave motion mixer (Heidolph, Schwbach, Germany, Polymax 1040). The plate was then read at an excitation of 360 nm and an emission of 440 nm using the VICTOR Nivo™ (PerkinElmer, Waltham, MA, USA, HH3522019094).

### 4.4. Examination of Calcium Deposition through Alizarin Red S Staining Morphology 

A key marker indicating the differentiation of ADMSCs into osteoblast lineage cells is the accumulation of calcium within the extracellular matrix, which can be visualized using Alizarin red S staining. Alizarin red S forms a complex with calcium ions, resulting in a vivid red stain that clearly highlights calcium deposits within the cellular matrix. For the calcium deposition assay, immortalized ADMSCs were seeded at a concentration of 1 × 10^4^ cells/mL in 200 µL of a complete osteogenic differentiation medium. The cells were encapsulated within a 10 µL fast-dextran hydrogel disc and cultured in BRAND^®^ 96-well strip plates (Sigma-Aldrich^®^, BR782306). Following the differentiation period, the cells were washed three times with 150 µL of PBS, which were then carefully pipetted to ensure a thorough removal of medium residues. The cells were then fixed in 150 µL of 4% formaldehyde for 15 min at room temperature to preserve cell morphology and matrix components. After fixation, the cells were washed three times with 150 µL of deionized water to remove any residual formaldehyde. The cells were then stained with 150 µL of 40 mM Alizarin red S (Sigma-Aldrich^®^, A5533) solution for 25 min at room temperature, ensuring that the staining solution thoroughly covered the cell surface. Post-staining, the cells underwent six washes with 150 µL of PBS to remove excess staining, minimizing background interference. The stained cells were then observed under an inverted light microscope (Olympus, Tokyo, Japan, CKX41), and images were captured using a digital camera (Olympus, SC30) linked to the microscope. The Olympus CellSens Imaging Software program version 2.4 was used to document the presence of calcification deposits, and it is indicated by the appearance of bright red to orange dots within the matrix.

### 4.5. Analysis of Cellular Proliferation

Cellular proliferation was evaluated by quantifying the adenosine triphosphate (ATP) levels within the cells. Adenosine triphosphate, essential for energy transfer in metabolically active cells, serves as an indicator of cellular proliferation. The CellTiter-Glo^®^ 2.0 ATP luminescence assay (Promega, G9241) was utilized for this purpose. This assay employs luciferase to produce a stable luminescent signal and prevent the release of endogenous ATP during cell lysis. In a Corning^®^ 96-well solid polystyrene microplate (Sigma-Aldrich^®^, CLS3912), 50 µL of CellTiter-Glo^®^ 2.0 reagent was added to an equal volume of cell suspension. The microplate was gently mixed at 25 rpm on a wave motion mixer (Heidolph, Polymax 1040) for 5 min to induce cell lysis, followed by incubation in the dark at room temperature for 10 min to stabilize the luminescent signal. Subsequently, the microplate was placed into the VICTOR Nivo™ Multimode Plate Reader (PerkinElmer, HH3522019094) to measure the luminescent signal in relative light units (RLUs). A well containing a complete medium without cells served as an experimental control, with the signal from the control well subtracted from that of the sample wells.

### 4.6. Cellular Survivability Determination

The MTT assay (Sigma-Aldrich^®^, TOX1) serves to evaluate cellular viability by measuring cellular metabolic activity. This colorimetric method hinges on the conversion of the yellow tetrazolium salt, MTT (3-(4,5-dimethylthiazol-2-yl)-2,5-diphenyltetrazolium bromide), by metabolically active cells into purple formazan crystals, which can be dissolved to yield a measurable colored solution. Cells were seeded at a concentration of 1 × 10^4^ cells/mL into 96-well cell culture microplates (Sigma-Aldrich^®^, BR782306). A reconstituted MTT labeling reagent (Sigma-Aldrich^®^, M-5655), constituting 10% of the culture medium volume, was added, followed by a 3 h incubation in a humidified atmosphere of 5% CO_2_ at 37 °C. A MTT solubilization solution (Sigma-Aldrich^®^, M-8910), matching the original culture medium volume, was then added to each well and gently mixed for 2 min on a wave motion mixer (Heidolph, Polymax 1040) at 25 rpm to aid in dissolving the MTT formazan crystals. Absorbance readings were taken at 570 nm using the VICTOR Nivo™ (PerkinElmer, HH3522019094) in a flat-bottomed Corning^®^ 96-well clear polystyrene microplate (Sigma-Aldrich^®^, CLS3370). A blank containing complete media without cells served as an experimental control, with the signal from the control well subtracted from that of the sample wells during statistical analysis.

### 4.7. LDH Assay Membrane Permeability Examination

When a cell membrane is compromised, LDH is released into the cytosol, indicating cytotoxicity. The CytoTox96^®^ Non-Radioactive Cytotoxicity Assay (Promega, G1780) utilizes an NADH-dependent method to convert tetrazolium salt into a spectrophotometrically measurable red formazan product. The amount of formazan generated correlates directly with the number of treated cells that are damaged or undergoing cell death. Cytotoxicity was assessed by adding equal volumes of 50 µL of a reconstituted reagent to culture media in flat-bottomed Corning^®^ 96-well clear polystyrene microplates (Sigma-Aldrich^®^, CLS3370), followed by incubation in darkness at room temperature for 30 min. Subsequently, the colorimetric compound was measured photometrically at 490 nm using the VICTOR Nivo™ Multimode Plate Reader (PerkinElmer, HH3522019094).

**Figure 6:**
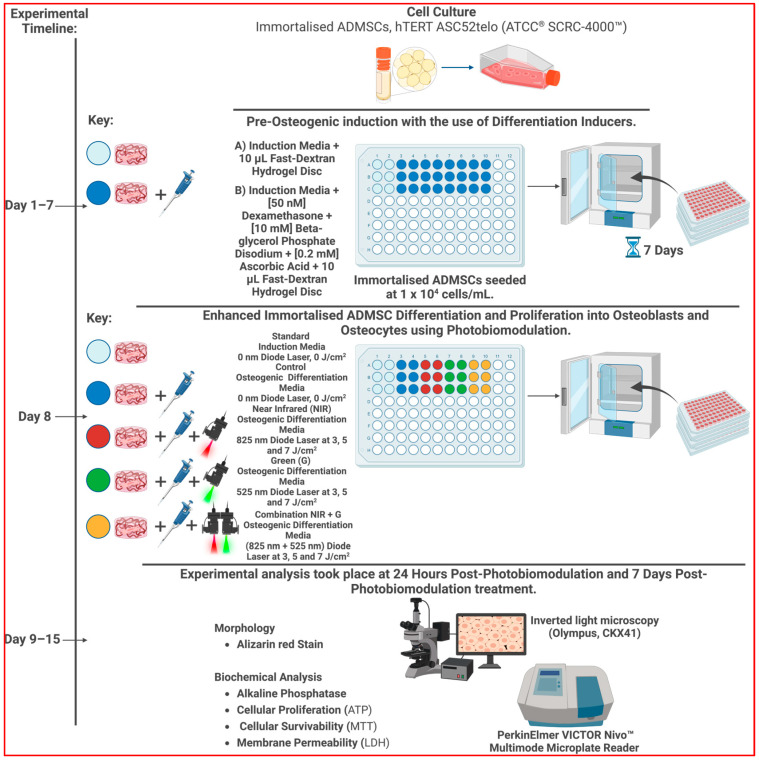
The experimental procedure: Immortalized adipose-derived stem cells were revived and sub-cultured until reaching the desired density. Osteogenic differentiation was induced using dexamethasone, β-glycerol phosphate disodium, and ascorbic acid within a fast-dextran hydrogel disc over several days of incubation. The cells were then exposed to different fluences (3 J/cm^2^, 5 J/cm^2^, and 7 J/cm^2^) at wavelengths of Near-Infrared 825 nm, Green 525 nm, and a combination thereof to enhance both osteoblastic differentiation and cellular proliferation. Two experimental conditions were tested: one where cells within the hydrogel disc received no osteogenic inducers or photobiomodulation treatment, and another where cells embedded within the hydrogel disc received only osteogenic inducers without photobiomodulation. Cell samples were collected at 24 h and 7 days post-irradiation. Alkaline phosphatase levels were quantified using spectrophotometry as an early protein marker, and calcium deposits were visualized using Alizarin red S staining. Biochemical analyses included ATP cell proliferation, MTT cell viability, and LDH membrane permeability assessments.

The methods of this in vitro study are illustrated in Figure 6.

### 4.8. Analytical Evaluation

For the statistical evaluation, experiments were conducted with triplicate biological repeats and technical duplicates. Spectrometry experiments involved subtracting the blank sample from the collected data. Statistical analysis was performed using SigmaPlot version 12, with error bars indicating the median (SEM) (n = 3). Normality was tested using either the Shapiro–Wilk or Kolmogorov–Smirnov tests before applying statistical analyses, including the Student t-test and one-way ANOVA. Statistical significance among experimental groups was represented on the figures as *p* < 0.05 (*), *p* < 0.01 (**), and *p* < 0.001 (***). Significant comparisons between experimental groups and the standard were denoted with a black star (*), comparisons between PBM groups and the control were marked with a blue star (*), and comparisons among experimental PBM groups were highlighted with a red star (*). Data from Alizarin red S staining were quantitatively analyzed using Image J, a freely available Java-based image processing program (National Institute of Health, Bethesda, MD, USA).

## 5. Conclusions

In conclusion, our study demonstrates the significant impact of PBM on the differentiation and proliferation of hydrogel-embedded immortalized ADMSCs into osteoblast-like cells. Alkaline phosphatase protein analysis consistently identified the G wavelength at 7 J/cm^2^ as the most effective PBM parameters for influencing ADMSC differentiation. Morphological investigations revealed the prominent calcium deposition in cells treated with G light (525 nm) at a fluency of 7 J/cm^2^. However, the NIR (825 nm) and NIR-G combination PBM treatment groups, regardless of fluency, showed similarly improved differentiation effects, as evidenced by the ATP proliferation, MTT viability, and LDH membrane permeability analyses. Further research is needed to pinpoint the optimal wavelength and fluency for maximizing ADMSC differentiation and proliferation into osteoblast-like cells. Despite this, our findings add to the combined use of embedded ADMSCs in 3D hydrogel matrices and PBM as a promising approach in regenerative medicine. These advancements pave the way for innovative treatments for degenerative bone diseases.

## Figures and Tables

**Table 1:** Parameters for laser exposure.

Laser	Near-Infrared (NIR)	Green (G)
**Light Source**	Diode laser	Diode laser
**Wavelength (nm)**	825	525
**Power Output (mW)**	187	551
**Spot Size (cm^2^)**	9.52	9.52
**Power Density (mW/cm^2^)**	20.60	60.68
**Intensity (W/cm^2^)**	0.02	0.06
**Emission**	Continuous wave	Continuous wave
**Fluence (J/cm^2^)**	3, 5 and 7	3, 5 and 7
**Time of Irradiation (s)**	145, 242 and 339	49, 82 and 115

## Data Availability

The data that support the findings of this study are available from the corresponding authors upon reasonable request.
